# Development and Internal Validation of Nomograms for Survival of Advanced Epithelial Ovarian Cancer Based on Established Prognostic Factors and Hematologic Parameters

**DOI:** 10.3390/jcm13102789

**Published:** 2024-05-09

**Authors:** Sherin Abdo Said, Joanna IntHout, Judith E. den Ouden, Janneke E. W. Walraven, Maaike A. van der Aa, Joanne A. de Hullu, Anne M. van Altena

**Affiliations:** 1 Department of Obstetrics and Gynecology, Radboud University Medical Center, 6525 GA Nijmegen, The Netherlandsanne.vanaltena@radboudumc.nl (A.M.v.A.); 2 Department of Research and Development, Netherlands Comprehensive Cancer Organization (IKNL), 3512 CV Utrecht, The Netherlands; 3 Department for Health Evidence, Radboud University Medical Center, 6525 GA Nijmegen, The Netherlands; 4 Department of Medical Oncology, Radboud University Medical Center, 6525 GA Nijmegen, The Netherlands

**Keywords:** epithelial ovarian cancer, anemia, leukocytosis, thrombocytosis, overall survival, prediction models

## Abstract

**Objective:** To assess the association between pretreatment thrombocytosis, anemia, and leukocytosis and overall survival (OS) of advanced-stage EOC. Furthermore, to develop nomograms using established prognostic factors and pretreatment hematologic parameters to predict the OS of advanced EOC patients. **Methods**: Advanced-stage EOC patients treated between January 1996 and January 2010 in eastern Netherlands were included. Survival outcomes were compared between patients with and without pretreatment thrombocytosis (≥450,000 platelets/µL), anemia (hemoglobin level of <7.5 mmol/L), or leukocytosis (≥11.0 × 10^9^ leukocytes/L). Three nomograms (for ≤3-, ≥5-, and ≥10-year OS) were developed. Candidate predictors were fitted into multivariable logistic regression models. Multiple imputation was conducted. Model performance was assessed on calibration, discrimination, and Brier scores. Bootstrap validation was used to correct for model optimism. **Results**: A total of 773 advanced-stage (i.e., FIGO stages IIB–IV) EOC patients were included. The median [interquartile range, IQR] OS was 2.3 [1.3–4.2] and 3.0 [1.4–7.0] years for patients with and without pretreatment thrombocytosis (*p* < 0.01). The median OS was not notably different for patients with and without pretreatment leukocytosis (*p* = 0.58) or patients with and without pretreatment anemia (*p* = 0.07). The final nomograms comprised established predictors with either pretreatment leukocyte or platelet count. The ≥5- and ≥10-year OS models demonstrated good calibration and adequate discrimination with optimism-corrected *c*-indices [95%-CI] of 0.76 [0.72–0.80] and 0.78 [0.73–0.83], respectively. The ≤3-year OS model demonstrated suboptimal performance with an optimism-corrected c-index of 0.71 [0.66–0.75]. **Conclusions**: Pretreatment thrombocytosis is associated with poorer EOC survival. Two well-performing models predictive of ≥5-year and ≥10-year OS in advanced-stage EOC were developed and internally validated.

## 1. Introduction 

Epithelial ovarian cancer (EOC) is the leading cause of death from gynecologic cancers in the western world [1]. In 2020, approximately 314,000 new cases of EOC and 207,000 EOC-related deaths were reported worldwide [2]. EOC predominantly affects postmenopausal women. The symptoms are nonspecific, such as abdominal fullness or distension, bloating, early satiety, nausea, fatigue, change in bowel movements, urinary symptoms, back pain, or unintended weight loss [3]. The diagnosis is based on gynecologic physical examination, transvaginal ultrasound, and the measurement of cancer antigen 125 (CA-125) level. Histologically, there are four main subtypes: serous, endometrioid, clear cell, and mucinous tumors [3,4]. Due to the nonspecific symptoms and the lack of effective screening tools for early detection, most patients are diagnosed at an advanced stage, i.e., International Federation Gynecology and Obstetrics (FIGO) stages IIB–IV [3]. In advanced-stage EOC, standard treatment includes cytoreductive surgery combined with platinum/taxane-based chemotherapy [5]. In the recent decade, the antiangiogenetic agent bevacizumab and poly(ADP-ribose) polymerase (PARP) inhibitors have been introduced as maintenance therapy for advanced-stage EOC [4]. While most patients achieve complete remission, 60–80% experience disease relapse and often succumb to the disease within 5 years after being diagnosed [6,7]. Nevertheless, a subgroup of patients may be long-term survivors, beyond 5–10 years [7,8,9,10,11,12,13]. This may depend on multiple factors including FIGO stage, age, histologic subtype, tumor grade, performance status, or residual disease. 

In addition to more established prognostic factors for EOC, there has been accumulating evidence on the prognostic value of high platelet count levels (i.e., preoperative thrombocytosis) in EOC [14,15,16]. Specifically, malignant EOC cells were demonstrated to produce thrombopoietic cytokines (i.e., IL-6) that lead to paraneoplastic thrombocytosis, which in turn contributes to tumor growth and metastatic development or growth [15,17,18]. Pretreatment thrombocytosis was associated with extensive initial disease burden, macroscopic residual disease after cytoreductive surgery, postoperative morbidity, and shortened survival [15,16,17,19]. Similarly, pretreatment leukocytosis and anemia, being linked to cancer progression, were also poor prognostic factors for EOC patients [15,20,21]. However, prior studies evaluating pretreatment anemia, leukocytosis, and thrombocytosis in EOC presented limited cohort sizes or clinical data. Therefore, it remains unclear whether these easily available parameters could really aid in the survival prediction of individual advanced-stage EOC patients in clinical practice. 

The aim of this study was to assess whether the aforementioned pretreatment hematologic parameters are associated with overall survival (OS) of advanced-stage EOC patients. In addition, the aim was to develop and internally validate three models predictive of ≤3-, ≥5-, and ≥10-year OS in advanced-stage EOC where established prognostic factors and pretreatment hematologic parameters are considered as predictors. These nomograms may be helpful for clinicians to estimate patients’ probabilities of ≤3-year, ≥5-, and ≥10-year OS. 

## 2. Materials and Methods 

### 2.1. Data Collection

Patients who underwent treatment for advanced-stage EOC (i.e., FIGO stages IIB–IV) between January 1996 and January 2010 in the eastern part of the Netherlands were selected. These patients were identified through a multicenter database that covers 1554 EOC patients from eleven participating Dutch hospitals and were selected since the time after their date of diagnosis exceeded 10 years. Extensive data on patients’ tumor and treatment characteristics were previously collected from patients’ medical records for registration and research purposes [22]. Survival data of the patients were obtained through the Netherlands Cancer Registry (NCR). The NCR is a nationwide cancer registry that is annually linked with municipality registries to update patients’ mortality status. 

### 2.2. Study Population 

Patients diagnosed with FIGO stages IIB up to IV EOC were identified. Patients who underwent cytoreductive surgery and received at least one cycle of platinum-based (neo-)adjuvant chemotherapy as part of their EOC treatment were included to ensure the study population underwent adequate treatment with a curative intent, enabling a proper assessment of the association between pretreatment hematologic parameters and overall survival of EOC.

### 2.3. Definitions 

Pretreatment thrombocytosis was defined as a platelet count of ≥450,000 platelets per microliter (consistent with Stone et al. who demonstrated a significant association between thrombocytosis and shortened survival [17]). Pretreatment anemia was defined as a hemoglobin level of <7.5 mmol per liter [23,24]. Pretreatment leukocytosis was defined as a leukocyte count of ≥11.0 × 10^9^ per liter [15,20]. Treatment approach was defined as primary cytoreductive surgery (PCS) followed by adjuvant chemotherapy, or neo-adjuvant chemotherapy followed by interval cytoreductive surgery and adjuvant chemotherapy (NACT-ICS). Platinum-based chemotherapy is generally initiated within six weeks after diagnosis and/or cytoreductive surgery. In addition, patients who were scheduled to undergo primary cytoreductive surgery, however, for whom the procedure was aborted, after which they received platinum-based chemotherapy and cytoreductive surgery, were considered NACT-ICS patients. Residual disease was defined as the maximum diameter of the largest tumor nodule remaining after cytoreductive surgery (classified as no macroscopic disease (complete cytoreduction) and macroscopic disease of ≤1 cm or >1 cm (optimal or incomplete cytoreduction)). 

### 2.4. Statistical Analysis and Software

Clinicopathologic characteristics were summarized using descriptive statistics. The OS was calculated as the time between the date of diagnosis and the date of death, or the date of last follow-up for patients who were still alive (31 January2023). To assess whether pretreatment anemia, leukocytosis, or thrombocytosis were associated with OS, Kaplan–Meier survival curves and log-rank tests were used. For the log-rank tests, the Kaplan–Meier survival curves were censored at the ten-year follow-up. Characteristics were demonstrated for the entire study population and patients with ≤3-, ≥5-, and ≥10-year OS. The cutoff point of ≤3-year OS was selected since the median OS of advanced-stage EOC patients is estimated at ~36 months [25]. The ≥5-year OS was selected to facilitate comparison with similar studies and FIGO reports [26,27,28,29]. Lastly, the ≥10-year OS was selected as a cutoff point for exceptionally long-term survival of advanced-stage EOC [8,9,12,13]. The transparent reporting of multivariable prediction models for individual prognosis or diagnosis (TRIPOD) guidelines were followed to report this study [30]. All statistical analyses were performed using STATA/SE, version 17.0 and R (version 4.0.3) (http://www.r-project.org) [31,32]. The following R packages were used for the analyses: “Hmisc” (version 4.7.0), “rms” (version 6.3.0), and “caret” (version 6.0.93) [33,34,35,36]. 

### 2.5. Model Development 

Three prediction models were developed and internally validated using the seven steps outlined in Steyerberg et al. [37]. The models were developed to predict probabilities of ≤3-, ≥5-, and ≥10-year OS. Candidate predictors considered included nine established prognostic factors (i.e., age at diagnosis, FIGO stage, tumor grade, histologic subtype, pretreatment CA-125 level, Karnofsky score, ascites volume, treatment approach, and residual disease after debulking) along with the following pretreatment hematological parameters: pretreatment hemoglobin level, platelet and leukocyte count, both as continuous and dichotomous variables. Continuous variables were transformed using logarithmic transformations when required. Multiple imputation was conducted using 30 imputations and 200 iterations. Candidate predictors were fitted into multivariable logistic regression models. Predictors were selected using backward selection (*p* < 0.50) to avoid using noise predictors in the models [38]. The results were pooled using Rubin’s rule [39]. Model performance was assessed on discrimination, calibration, and Brier scores.

I. Discrimination, i.e., the model’s ability to distinguish between patients with and without the survival outcome of interest, was assessed using the Harrell’s concordance (c)-index [40]. A value of 0.5 indicates that the model is no better than predicting an outcome than random chance. Conversely, a value of 1 indicates that the model perfectly predicts who will experience a certain outcome from those who will not.
II. Calibration, i.e., the agreement between the predicted and observed rates on a (sub)group level, was assessed with calibration plots, calibration intercepts, and slopes.
III. The Brier score is an overall performance measure calculated as the mean (squared) difference between the observed and the predicted outcomes. The lower the score, the better the predictions reflect the observed data. A score near 0 indicates perfect accuracy.

### 2.6. Model Validation 

Internal validation was performed using the boot-MI method as proposed by Bartlett and Hughes [39]. A total of 100 bootstrap samples were drawn from the development sample. The entire model development process, including multiple imputation, was repeated in each bootstrap sample. Bootstrapping was used to estimate and correct for optimism in *c*-indices, calibration, and the Brier scores and to estimate shrinkage factors for the final models. After internal validation, the shrinkage factors were used to re-estimate the regression coefficients and model intercepts. 

### 2.7. Ethical Approval

Ethical approval from the NCR’s Committee of Privacy was acquired for this study [K17-245]. 

## 3. Results

### 3.1. Study Population

**Figure 1:**
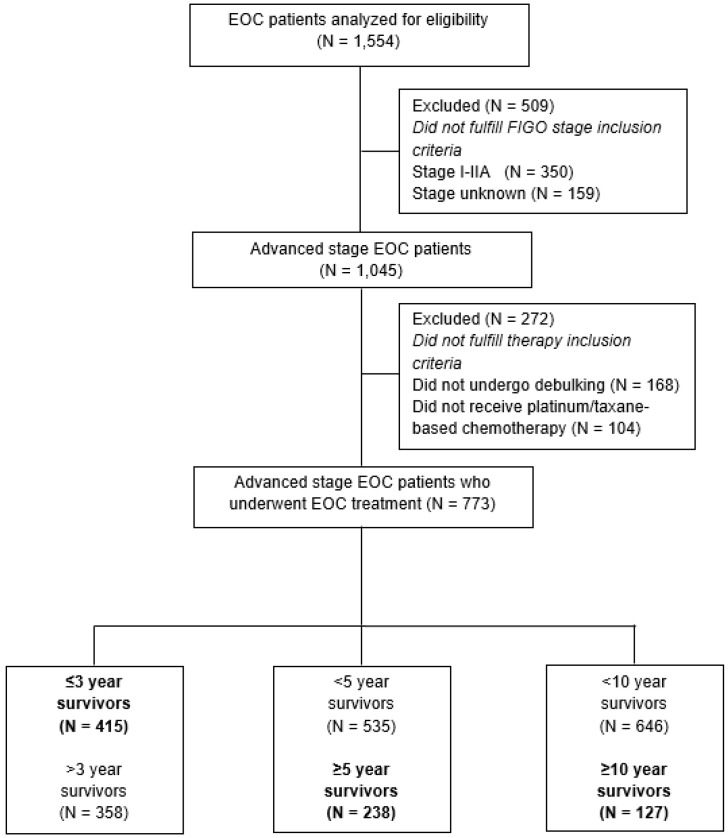
Flow chart of the study population.

A total of 1045 patients were diagnosed with advanced-stage EOC between January 1996 and January 2010 in the eastern part of the Netherlands (Figure 1). Of these patients, 773 patients underwent cytoreductive surgery in combination with platinum-based chemotherapy (i.e., PCS or NACT-ICS). Overall, 415/773 patients survived ≤3 years (53.7%), 238/773 (30.8%) survived ≥5 years, and 127/773 (16.4%) survived ≥10 years. 

The patient, tumor, and treatment characteristics are summarized in Table 1. The ≤3-year survivors were slightly older than the ≥5- and ≥10-year survivors. In addition, the ≤3-year survivors consisted of relatively more patients with FIGO stages IIIC and IV and less patients with FIGO stages IIB–IIIB. The serous type of EOC was the most common histologic subtype among the ≤3-, ≥5-, and ≥10-year survivors. However, the ≥5- and ≥10-year survivors comprised relatively more patients with the endometrioid type of EOC than the ≤3-year survivors. Moreover, the ≤3-year survivors consisted of more patients with Karnofsky scores of 50 up to 70 and less patients with 80 up to 100 than the ≥5- and ≥10-year survivors. The ≤3-year survivors also comprised more patients with pretreatment thrombocytosis compared with the ≥5- and ≥10-year survivors. Similarly, the ≤3-year survivors comprised a slightly higher proportion of patients with preoperative anemia and leukocytosis than the ≥5- and ≥10-year survivors. Lastly, the ≤3-year survivors comprised relatively less patients who underwent PCS or complete cytoreduction compared with the ≥5- and ≥10-year survivors. 

### 3.2. OS and Pretreatment Hematologic Parameters 

**Figure 2:**
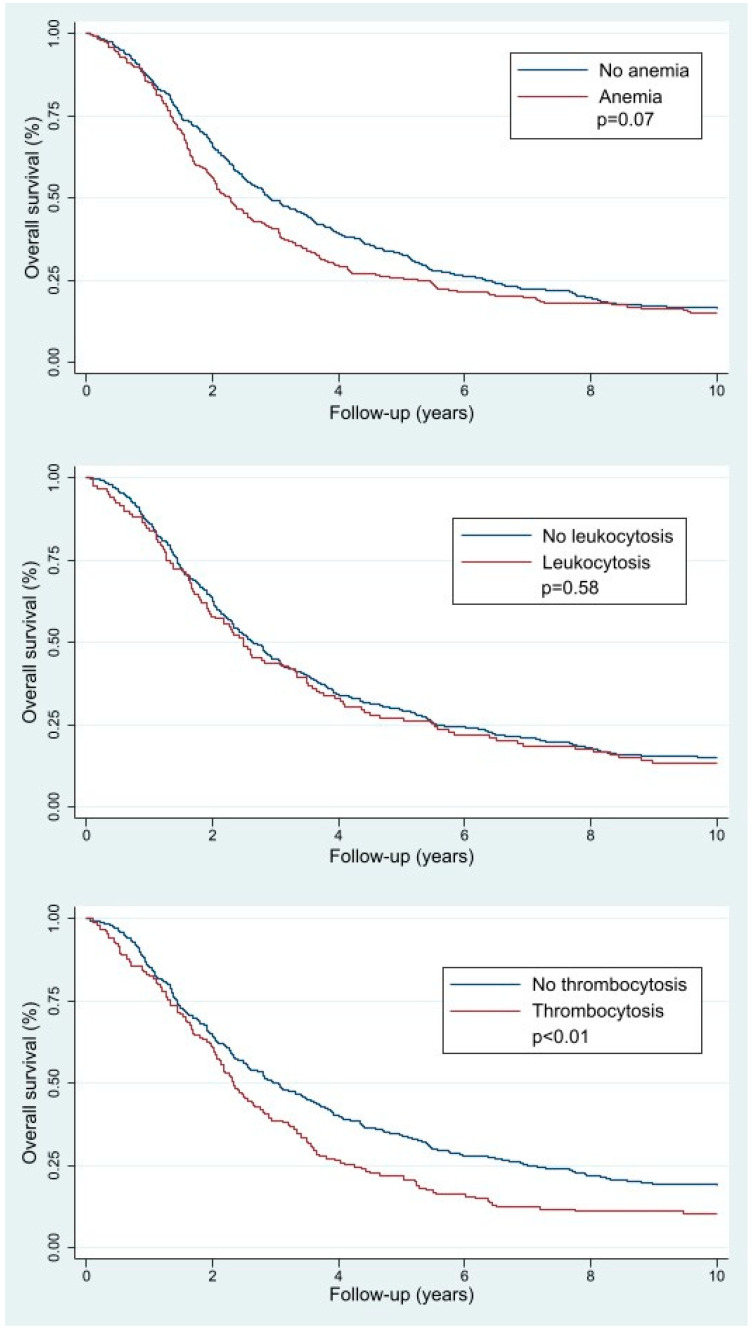
Kaplan–Meier survival curves for the overall survival of the different pretreatment hematologic parameter subgroups. The patients with pretreatment anemia (N = 225), leukocytosis (N = 119), or thrombocytosis (N = 155) are demonstrated in red, whereas patients without pretreatment thrombocytosis (N = 389), leukocytosis (N = 461), or anemia (N = 505) are demonstrated in blue. The *p*-values are provided at the different Kaplan–Meier survival curves.

Figure 2 demonstrates the Kaplan–Meier survival curves used to calculate the median OS for the patients with and without pretreatment thrombocytosis, leukocytosis, and anemia. The median [IQR] OS was 3 [1.4–7.0] years for the patients without pretreatment thrombocytosis compared with 2.3 [1.3–4.2] years for the patients with pretreatment thrombocytosis (*p* < 0.01). Furthermore, the median [IQR] OS was 2.7 [1.4–5.6] years for the patients without pretreatment leukocytosis compared to 2.5 [1.3–5.5] years for the patients with pretreatment leukocytosis (*p* = 0.58). In addition, median [IQR] OS was 2.9 [1.5–6.3] years for the patients without pretreatment anemia compared to a median [IQR] OS of 2.3 [1.4–5.3] years for the patients with pretreatment anemia (*p* = 0.07).

### 3.3. Final Prediction Models and Their Parameters

After the variable selection processes, the three prediction models comprised different sets of predictors. The most predictive ≤3-year OS model contained pretreatment leukocyte count, age at diagnosis, FIGO stage, tumor grade, histologic subtype, Karnofsky score, ascites volume, treatment approach, and residual disease after debulking. The most predictive ≥5-year OS model included the same predictors as the ≤3-year OS model but excluding tumor grade and histologic subtype as predictors. Lastly, the ≥10-year OS model included pretreatment platelet count, FIGO stage, tumor grade, Karnofsky score, treatment approach, and residual disease after debulking. The final OS models are listed in Supplementary Tables S1–S3.

### 3.4. Model Performance 

The *c*-indices of the ≤3-year, ≥5-year, and ≥10-year OS prediction models were estimated at 0.74, 0.78, and 0.82, respectively. Additionally, the Brier scores were estimated at 0.21, 0.17, and 0.11, respectively. The calibration plots of all models showed that the calibration curves of the different models were close to the perfect fit line (see Supplementary Figures S1–S3). 

### 3.5. Internal Validation 

Internal validation using 100 bootstrap iterations estimated the optimism-corrected *c*-indices at 0.71 [95%-CI 0.66–0.75], 0.76 [95%-CI 0.72–0.80], and 0.78 [95%-CI 0.73–0.83] for the ≤3-year, ≥5-year, and ≥10-year OS models, respectively. In addition, the Brier scores were re-estimated at 0.22 [95%-CI 0.20–0.23], 0.18 [95%-CI 0.17–0.19], and 0.12 [95%-CI 0.10–0.13], respectively. The optimism-corrected calibration slopes (i.e., shrinkage factors) were estimated to be 0.85 [95%-CI 0.82–0.88], 0.87 [95%-CI 0.85–0.89], and 0.82 [95%-CI 0.79–0.86], respectively. These shrinkage factors were used to re-estimate the regression coefficients and intercepts of the respective final shrunken models. The final OS models and the coefficients of the included parameters before and after internal validation are listed in Supplementary Tables S1–S3. 

### 3.6. Risk Stratification 

Risk stratification tables show the sensitivities, specificities, positive and negative predictive values, and the positive likelihood ratios according to different cutoffs for the predicted probabilities of the final prediction models. Predicted probabilities greater than or equal to the cutoff are defined to be fulfilling the prediction to survive at least 10 years. Table 2 shows that when the cutoff for patients’ probability of ≥10-year OS is set at 25%, the final ≥10-year OS model has a sensitivity of 55.9%, a specificity of 87.5%, and a positive and negative predictive value of 46.7% and 91.0%, respectively. The risk stratification table of the final ≥5-year OS model is demonstrated in Supplementary Table S4.

### 3.7. Nomogram

**Figure 3:**
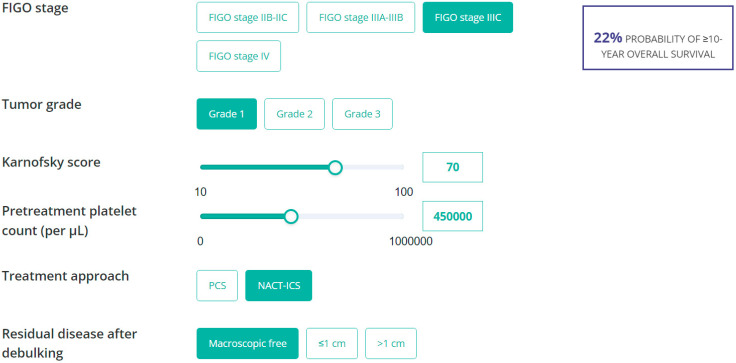
Snapshot of the online score calculator for the ≥10-year OS model. The online score calculator allows clinicians to estimate the probability of ≥10-year overall survival. For example, for a patient with FIGO stage IIIC EOC who presented with a low-grade tumor, Karnofsky score of 70, pretreatment platelet count of 450,000 per µL, and who underwent NACT-ICS with complete cytoreduction, their nomogram predicts a probability of 22% of ≥10-year OS for this patient.

Online score calculators were built using the internally validated estimates of the final ≥5- or ≥10-year OS models and are freely accessible at Evidencio.com (link 1, link 2). To calculate the probabilities of ≥5-year or ≥10-year OS for an advanced-stage EOC patient who underwent cytoreductive surgery, each calculator requires the relevant parameter values of that patient. An example of the online nomogram that predicts the probability of ≥10-year OS is demonstrated in Figure 3. An example of the online score calculator of the ≥5-year model is demonstrated in Supplementary Figure S4. 

## 4. Discussion

In this population-based study, the prognostic value of three pretreatment hematological parameters (i.e., pretreatment anemia, leukocytosis, and thrombocytosis) was assessed. Our data confirm that pretreatment thrombocytosis is associated with worse overall survival of advanced-stage EOC. No significant association was found between pretreatment anemia or leukocytosis and overall survival. In addition, three nomograms were developed and internally validated using established prognostic factors along with either pretreatment leukocyte count or platelet count as predictors.

Online score calculators were built for the models that predict the probabilities of ≥5- or ≥10-year OS for individual advanced-stage EOC patients on a freely accessible online platform (Evidencio.com).

Pretreatment thrombocytosis was shown to be associated with higher initial disease burden, postoperative morbidity, disease progression, and decreased OS of EOC [14,15,16,17,18,19,20,41,42]. Our data confirm this last finding. This might further support the theory that high platelet counts at diagnosis contribute to tumor or metastatic growth, which could hamper patients from demonstrating long-term survival. Accordingly, pretreatment platelet count was selected as a useful predictor in the ≥10-year OS model. Specifically, patients who do not present with pretreatment thrombocytosis (i.e., patients with low or normal platelet counts) have a higher probability of long-term survival.

Furthermore, pretreatment anemia was linked with low performance status, chemotherapy delays, chemotherapy dose reductions, and decreased quality of life for cancer patients [43,44]. Our data did not show a significant difference in the OS of patients with pretreatment anemia than those without pretreatment anemia. Gerestein et al. (N = 118) incorporated pretreatment hemoglobin levels into their nomogram to predict probabilities of 5-year OS of advanced-stage EOC patients [28]. Despite demonstrating survival differences up to a follow-up of five years, our data did not show that preoperative anemia is significantly associated with overall survival. Pretreatment hemoglobin level was also not selected as a final predictor in either of our three final OS models since other combinations of predictors resulted in better performing predictive models. The inclusion of pretreatment hemoglobin level in the model of Gerestein et al. is likely due to the slightly different combination of candidate predictors (e.g., albumin and lactate dehydrogenase levels) incorporated in their model or a different study population. Nevertheless, the *c*-index of their nomogram was estimated at 0.67 (0.62 at external validation) compared with a higher *c*-index of 0.76 for our ≥5-year OS model [28].

Contrary to the two aforementioned hematologic parameters, the prognostic value of pretreatment leukocytosis in advanced-stage EOC remains unclear due to inconsistent findings in the literature [21,45,46]. For instance, So et al. demonstrated an independent association between pretreatment leukocytosis and shortened PFS and OS. Their study (N = 155) was solely based on patients who underwent primary cytoreductive surgery [21]. Chen et al. (N = 816), on the other hand, did not demonstrate an independent association between pretreatment leukocytosis and decreased EOC survival [15]. In line with Chen et al., our data did not demonstrate a difference in median OS of patients with or without pretreatment leukocytosis. Nevertheless, preoperative leukocyte count did add to the prediction of ≤3-year and ≥5-year OS for advanced-stage EOC patients.

Several prognostic nomograms have been developed for predicting EOC survival [26,27,28,29,47,48]. However, most of these nomograms did not include patients who underwent NACT-ICS (except Rutten et al.) [27,28,29,48,49]. In addition, existing models predominantly focus on the 5-year OS of EOC patients and do not provide predictions of the ≤3-year and ≥10-year OS of advanced-stage EOC patients. The inclusion of advanced-stage EOC patients, encompassing all histologic subtypes and undergoing NACT-ICS or PCS combined with platinum/taxane-based chemotherapy in our models, enhances the generalizability of our findings to a broader population of EOC patients. Although external validation of the models is required, our prognostic nomograms are expected to be inexpensive and readily applicable tools for obtaining more reliable prognostic information for individual advanced-stage EOC patients after cytoreductive surgery than the current models that are available. In addition to more individualized patient counseling on prognosis, these nomograms may be useful in postoperative counseling of patients and perhaps in the assessment of patient eligibility for clinical trials.

Regarding the limitations of our study, it is essential to acknowledge that the ≤3-year OS model exhibited inadequate performance, resulting in a high rate of patients being incorrectly classified as ≤3-year survivors. Therefore, this model is unsuitable for predicting the probability of ≤3-year OS. Furthermore, due to the retrospective nature of the data, the lack of sufficient data on other possible predictors (e.g., *BRCA* status, postoperative CA-125 level, CA-125 nadir, or the use of HIPEC) did not allow for these factors to be included in the model development. In addition, the data used in our study dated back to the era before PARP inhibitors. Therefore, PARP inhibitor usage could not be used as a potential predictor in the development of the current prediction models. Namely, different phase III trials (i.e., SOLO-1, PAOLA-1, PRIMA, and VELIA) demonstrated significant improvement in progression-free survival of advanced-stage EOC [4]. However, long-term overall survival data from these trials are still pending. Therefore, it is important to update the models when these data become available to assess their impact on patients’ survival.

## 5. Conclusions

In conclusion, pretreatment thrombocytosis is significantly associated with poorer EOC survival.

However, no significant association was observed between pretreatment anemia or leukocytosis and overall survival. Two adequate performing models were developed and internally validated to predict the probabilities of ≥5-year and ≥10-year OS for individual advanced-stage EOC patients.

## Figures and Tables

**Table 1:** Patient, tumor, and treatment characteristics of the study population (N = 773).

	Total N = 773 (%)/Median [IQR]	≤3-Year OS N = 415 (%)/Median [IQR]	≥5-Year OS N = 238 (%)/Median [IQR]	≥10-Year OS N = 127 (%)/Median [IQR]
Characteristic				
Age at diagnosis (in yrs)				
Median [IQR]	61 [21–84]	63 [28–84]	60 [27–80]	59 [38–77]
FIGO stage				
Stage IIB–IIC	83 (10.7)	16 (3.9)	61 (25.6)	48 (37.8)
Stage IIIA–IIIB	87 (11.3)	41 (9.9)	31 (13.0)	18 (14.2)
Stage IIIC	506 (65.5)	292 (70.4)	134 (56.3)	60 (47.2)
Stage IV	97 (12.5)	66 (15.9)	12 (5.0)	1 (0.8)
Tumor grade				
Grade 1	42 (5.4)	15 (3.6)	21 (8.8)	19 (15.0)
Grade 2	172 (22.3)	83 (20.0)	63 (26.5)	34 (26.8)
Grade 3	452 (58.5)	259 (62.4)	125 (52.5)	64 (50.4)
Unknown	107 (13.8)	58 (14.0)	29 (12.2)	10 (7.9)
Histologic subtype				
Serous	445 (57.6)	251 (60.5)	118 (49.6)	54 (42.5)
Mucinous	29 (3.8)	20 (4.8)	6 (2.5)	4 (3.2)
Endometrioid	92 (11.9)	40 (9.7)	41 (17.2)	27 (21.3)
Clear cell	23 (3.0)	12 (2.9)	9 (3.8)	8 (6.3)
Adenocarcinoma NOS *	146 (18.9)	72 (17.4)	51 (21.4)	28 (22.1)
Other	35 (4.5)	19 (4.6)	12 (5.0)	6 (4.7)
Unknown	3 (0.4)	1 (0.2)	1 (0.4)	0 (0)
Karnofsky score				
10–40	3 (0.4)	2 (0.5)	0 (0)	0 (0)
50–70	187 (24.2)	128 (30.8)	35 (14.7)	18 (14.2)
80–100	492 (63.7)	224 (54.0)	184 (77.3)	96 (75.6)
Unknown	91 (11.8)	61 (14.7)	19 (8.0)	13 (10.2)
Pretreatment CA-125 serum level (kU/L)				
Median [IQR]	484 [9–25,784]	666 [24–13,995]	334 [9–9219]	259 [10–4180]
Unknown	43 (5.6)	26 (6.3)	8 (3.4)	4 (3.1)
Pretreatment hemoglobin level (mmol/L)				
Median [IQR]	7.9 [4.6–9.9]	7.8 [4.6–9.6]	8.1 [5.7–9.7]	8.1 [5.9–9.7]
No anemia	505 (65.3)	257 (61.9)	167 (70.2)	82 (64.6)
Anemia	225 (29.1)	134 (32.4)	58 (24.4)	34 (26.8)
Unknown	43 (5.6)	24 (5.8)	13 (5.5)	11 (8.7)
Pretreatment platelet count (×10^3^/µL)				
Median [IQR]	370 [144–898]	390 [158–749]	336 [169–637]	324 [194–590]
No thrombocytosis	369 (47.7)	185 (44.6)	126 (52.9)	69 (54.3)
Thrombocytosis	155 (20.1)	95 (22.9)	34 (14.3)	16 (12.6)
Unknown	249 (32.2)	135 (32.5)	78 (32.8)	42 (33.1)
Pretreatment leukocyte count (×10^9^/L)				
Median [IQR]	8.4 [3.6–20.2]	8.6 [4.5–16.8]	8.1 [4–17.8]	8.3 [4.6–14.8]
No leukocytosis	461 (59.6)	255 (61.5)	136 (57.1)	68 (53.5)
Leukocytosis	119 (15.4)	67 (16.2)	32 (13.5)	16 (12.6)
Unknown	193 (25.0)	93 (22.8)	70 (29.4)	43 (33.9)
Presence of ascites				
No	142 (18.4)	46 (11.1)	75 (31.5)	45 (35.4)
Yes	608 (78.7)	355 (84.5)	158 (66.4)	80 (63.0)
Unknown	23 (3.0)	14 (3.4)	5 (2.1)	2 (1.6)
Ascites volume (mL)				
Median [IQR]	700 [0–18,000]	2000 [0–14,000]	100 [0–7000]	50 [0–6000]
Unknown	172 (22.2)	91 (22.0)	53 (22.2)	25 (19.7)
Treatment approach				
PCS	523 (67.7)	264 (63.6)	187 (78.6)	105 (82.7)
NACT-ICS	250 (32.3)	151 (36.4)	51 (21.4)	22 (17.3)
Residual disease after debulking				
No macroscopic disease	285 (36.9)	102 (24.6)	138 (58.0)	85 (66.9)
≤1 cm	265 (34.3)	153 (36.9)	70 (29.4)	31 (24.4)
>1 cm	186 (24.1)	137 (33.0)	22 (9.2)	8 (6.3)
Unknown	37 (4.8)	23 (5.4)	8 (3.4)	3 (2.4)

* The subcategory ‘adenocarcinoma NOS’ comprises the patients who had epithelial ovarian cancer without further specification on the histologic subtype of the epithelial ovarian cancer. The subcategories labeled ‘Unknown’ of the different variables refer to the unknown or missing data of that specific variable within the study cohort. Abbreviations: FIGO, International Federation of Gynecology and Obstretics; IQR, interquartile range; and NOS, not otherwise specified.

**Table 2:** Risk stratification table to assess the performance of the final ≥10-year overall survival model for different predicted probabilities ^a^.

Predicted Probabilities ^b^	Sensitivity (%)	Specificity (%)	PPV (%)	NPV (%)	LR+
≥5%	97.6	31.0	21.8	98.5	1.4
≥10%	88.2	55.6	28.1	96.0	2.0
≥15%	73.2	71.8	33.8	93.2	2.6
≥20%	62.2	83.3	42.2	91.8	3.7
≥25%	55.9	87.5	46.7	91.0	4.5
≥30%	48.0	91.3	52.1	89.9	5.5
≥35%	40.2	94.1	57.3	88.9	6.8
≥40%	37.0	95.2	60.2	88.5	7.7
≥45%	35.4	95.8	62.5	88.3	8.4
≥50%	33.1	96.3	63.6	88.0	8.9
≥55%	30.0	97.4	69.0	87.6	11.5
≥60%	23.6	98.0	69.8	86.7	11.8
≥65%	13.4	98.9	70.8	85.3	12.2
≥70%	6.3	99.7	80.0	84.4	21
≥75%	4.7	99.7	75.0	84.2	15.7
≥80%	3.9	100	100	84.1	-
≥85%	-	-	-	-	-
≥90%	-	-	-	-	-
≥95%	-	-	-	-	-
≥100%	-	-	-	-	-

Abbreviations: PPV, positive predictive value; NPV, negative predictive value; and LR+, positive likelihood ratio (calculated using the following equation: sensitivity/1-specificity). ^a^ Predicted probability of having ≥10-year OS. ^b^ Predicted probabilities of the final ≥10-year OS model.

## Data Availability

Restrictions apply to the availability of these data. Data were obtained from the Netherlands Cancer Registry and are available from S.A. Said with the permission of the Netherlands Cancer Registry.

## Supplementary Materials

The following supporting information can be downloaded at: https://www.mdpi.com/article/10.3390/jcm13102789/s1, Table S1. Final ≤3-year OS model (≤3 year survivors (N = 415) and >3 year survivors (N = 358)). Table S2. Final ≥5-year OS model (<5 year survivors (N = 535) and ≥5 year survivors (N = 238)). Table S3. Final ≥10-year OS model (<10 year survivors (N = 646) and ≥10 year survivors (N = 127)). Table S4. Risk stratification table to assess the performance of the final ≥5-year overall survival model at different predicted probabilities^a^. Figure S1. Calibration plot of the ≤3-year OS model before and after internal validation. The ideal line represents the perfect fit line. The model after backward selection represents the model before internal validation (green dotted line). The shrunken backward selection model represents the model after internal validation (blue line). The full model represents the model with all the candidate predictors (red line). The calibration plot demonstrates that the final ≤3-year OS model is well-calibrated. Figure S2. Calibration plot of the ≥5-year OS model before and after internal validation. The ideal line represents the perfect fit line. The model after backward selection represents the model before internal validation (green dotted line). The shrunken backward selection model represents the model after internal validation (blue line). The full model represents the model with all the candidate predictors (red line). The calibration plot demonstrates that the final ≥5-year OS model is well-calibrated. Figure S3. Calibration plot of the ≥10-year OS model before and after internal validation. The ideal line represents the perfect fit line. The model after backward selection represents the model before internal validation (green dotted line). The shrunken backward selection model represents the model after internal validation (blue line). The full model represents the model with all the candidate predictors (red line). The calibration plot demonstrates that the final ≥10-year OS model is well-calibrated. Figure S4. Screenshot of the online score calculator for the ≥5-year OS model. The online score calculator allows clinicians to estimate the probability of ≥5-year OS. For example: a 81-year-old patient with FIGO stage IIIC EOC, who presented with Karnofsky score of 70, pretreatment leukocytes count of 7 ×109 per liter, 500 mL of ascites volume, who underwent primary cytoreductive surgery and complete cytoreduction. The nomogram predicts a probability of 53% of ≥5-year OS for this patient.

## Abbreviations

EOC	Epithelial Ovarian Cancer
FIGO	International Federation of Gynecology and Obstretics
PCS	Primary Cytoreductive Surgery
NACT-ICS	Neo-Adjuvant Chemotherapy followed by Interval Cytoreductive Surgery
IL-6	Interleukin 6
OS	Overall Survival
CA-125	Cancer Antigen 125
NCR	Netherlands Cancer Registry
IQR	Interquartile Range
LR+	Positive Likelihood Ratio
PPV	Positive Predictive Value
NPV	Negative Predictive Value
CI	Confidence Interval
HIPEC	Hyperthermic Intraperitoneal Chemotherapy
PARP	Poly(ADP-ribose) Polymerase
