# Supplementary material for: Development and Internal Validation of Nomograms for Survival of Advanced Epithelial Ovarian Cancer Based on Established Prognostic Factors and Hematologic Parameters
Source: J Clin Med. 2024 May 9;13(10):2789. doi: 10.3390/jcm13102789 (PMC11122536; doi:10.3390/jcm13102789)
Supplement: Supplementary file 1 [file jcm-13-02789-s001.zip › jcm-2955976-supplementary.docx]

**Supplementary**

Supplementary tables

| **Supplementary table S1.** Final ≤3-year OS model (≤3 year survivors (N = 415) and >3 year survivors (N = 358)) | | | | |
| --- | --- | --- | --- | --- |
|  | Original model* | | Shrunken model* | |
| Characteristic | Odds ratios | 95% CI | Odds ratios | 95% CI |
| Age at diagnosis |  |  |  |  |
| <74 yrs | Reference |  | Reference |  |
| ≥75 yrs | 2.08 | 1.21-3.57 | 1.86 | 1.08-3.19 |
| FIGO stage |  |  |  |  |
| Stage IIB-IIC | Reference |  | Reference |  |
| Stage IIIA-IIIB | 2.31 | 1.10-4.85 | 2.03 | 0.97-4.27 |
| Stage IIIC | 2.46 | 1.27-4.74 | 2.14 | 1.10-4.13 |
| Stage IV | 3.88 | 1.76-8.53 | 3.15 | 1.43-6.93 |
| Tumor grade |  |  |  |  |
| Grade 1 | Reference |  | Reference |  |
| Grade 2 | 1.15 | 0.54-2.45 | 1.12 | 0.53-2.39 |
| Grade 3 | 1.61 | 0.79-3.31 | 1.50 | 0.73-3.08 |
| Histologic subtype |  |  |  |  |
| Serous | Reference |  | Reference |  |
| Non-serous | 1.29 | 0.86-1.95 | 1.24 | 0.83-1.87 |
| Adenocarcinoma NOS | 0.77 | 0.50-1.17 | 0.80 | 0.52-1.22 |
| Karnofsky score (per 10 points) |  |  |  |  |
|  | 0.82 | 0.69-0.96 | 0.84 | 0.72-0.99 |
| Pretreatment leukocytes count (ln) |  |  |  |  |
|  | 1.87 | 1.06-3.32 | 1.70 | 0.96-3.02 |
| Ascites volume (ln) |  |  |  |  |
|  | 1.06 | 1.01-1.13 | 1.05 | 1.00-1.11 |
| Treatment approach |  |  |  |  |
| PCS | Reference |  | Reference |  |
| NACT-ICS | 1.34 | 0.92-1.94 | 1.28 | 0.88-1.85 |
| Residual disease after debulking |  |  |  |  |
| Macroscopic free | Reference |  | Reference |  |
| ≤1 cm | 1.99 | 1.36-2.91 | 1.79 | 1.22-2.62 |
| >1 cm | 3.30 | 1.06-3.32 | 2.75 | 1.73-4.38 |
| Model intercept | 0.16 | 0.02-1.35 | 0.21 | 0.03-1.84 |
| Abbreviations: CI, confidence interval.  * The original model comprises the results before internal validation and the shrunken model comprises the results after internal validation where the shrinkage factor of 0.847 was used to shrink the coefficients. | | | | |

| **Supplementary table S2.** Final ≥5-year OS model (<5 year survivors (N = 535) and ≥5 year survivors (N = 238)) | | | | |
| --- | --- | --- | --- | --- |
|  | Original model* | | Shrunken model* | |
| Characteristic | Odds ratios | 95% CI | Odds ratios | 95% CI |
| Age at diagnosis |  |  |  |  |
| <74 yrs | Reference |  | Reference |  |
| ≥75 yrs | 0.54 | 0.28-1.03 | 0.58 | 0.31-1.12 |
| FIGO stage |  |  |  |  |
| Stage IIB-IIC | Reference |  | Reference |  |
| Stage IIIA-IIIB | 0.33 | 0.16-0.68 | 0.39 | 0.19-0.78 |
| Stage IIIC | 0.35 | 0.19-0.64 | 0.40 | 0.22-0.74 |
| Stage IV | 0.13 | 0.06-0.31 | 0.17 | 0.07-0.40 |
| Karnofsky score (per 10) |  |  |  |  |
|  | 1.28 | 1.07-1.54 | 1.24 | 1.03-1.49 |
| Pretreatment leukocytes count (ln) |  |  |  |  |
|  | 0.46 | 0.24-0.86 | 0.50 | 0.27-0.95 |
| Ascites volume (ln) |  |  |  |  |
|  | 0.92 | 0.87-0.98 | 0.93 | 0.88-0.99 |
| Treatment approach |  |  |  |  |
| PCS | Reference |  | Reference |  |
| NACT-ICS | 0.53 | 0.35-0.81 | 0.57 | 0.38-0.88 |
| Residual disease after debulking |  |  |  |  |
| Macroscopic free | Reference |  | Reference |  |
| ≤1 cm | 0.52 | 0.34-0.78 | 0.57 | 0.37-0.85 |
| >1 cm | 0.23 | 0.13-0.41 | 0.28 | 0.16-0.50 |
| Model intercept | 2.82 | 0.31-25.57 | 2.25 | 0.24-20.38 |
| Abbreviations: CI, confidence interval, natural log.  * The original model comprises the results before internal validation and the shrunken model comprise the results after internal validation where the shrinkage factor of 0.847 was used to shrink the coefficients. | | | | |

| **Supplementary table S3.** Final ≥10-year OS model (<10 year survivors (N = 646) and ≥10 year survivors (N = 127)) | | | | |
| --- | --- | --- | --- | --- |
|  | Original model* | | Shrunken model* | |
| Characteristic | Odds ratio (OR) | 95% CI | Odds ratio (OR) | 95% CI |
| FIGO stage |  |  |  |  |
| Stage IIB-IIC | Reference |  | Reference |  |
| Stage IIIA-IIIB | 0.29 | 0.14-0.59 | 0.35 | 0.17-0.73 |
| Stage IIIC | 0.25 | 0.14-0.46 | 0.32 | 0.17-0.58 |
| Stage IV | 0.02 | 0.00-0.17 | 0.04 | 0.01-0.33 |
| Tumor grade |  |  |  |  |
| Grade 1 | Reference |  | Reference |  |
| Grade 2 | 0.42 | 0.19-0.93 | 0.48 | 0.22-1.08 |
| Grade 3 | 0.37 | 0.17-0.77 | 0.44 | 0.21-0.92 |
| Pretreatment platelet counts (ln) |  |  |  |  |
|  | 0.81 | 0.66-1.00 | 0.84 | 0.68-1.03 |
| Karnofsky score (per 10 points) |  |  |  |  |
|  | 1.26 | 1.00-1.60 | 1.21 | 0.96-1.53 |
| Treatment approach |  |  |  |  |
| PCS | Reference |  | Reference |  |
| NACT-ICS | 0.58 | 0.33-1.03 | 0.64 | 0.36-1.13 |
| Residual disease after debulking |  |  |  |  |
| Macroscopic free | Reference |  | Reference |  |
| ≤1 cm | 0.42 | 0.25-0.71 | 0.49 | 0.29-0.82 |
| >1 cm | 0.21 | 0.09-0.46 | 0.27 | 0.12-0.61 |
| Model intercept | 1.01 | 0.10-9.96 | 0.81 | 0.08-7.95 |
| Abbreviations: CI, confidence interval; ln, natural log.  *The original model comprises the results before internal validation and the shrunken model comprise the results after internal validation where the shrinkage factor of 0.827 was used to shrink the coefficients. | | | | |
|  | | | | |

| **Supplementary table S4.** Risk stratification table to assess the performance of the final ≥5-year overall survival model at different predicted probabilities^a^ | | | | | |
| --- | --- | --- | --- | --- | --- |
| Predicted probability ^b^ | Sensitivity (%) | Specificity (%) | PPV (%) | NPV (%) | LR+ |
| ≥ 5% | 99.6 | 5.6 | 31.9 | 96.8 | 1.1 |
| ≥ 10% | 98.7 | 21.5 | 35.9 | 97.4 | 1.3 |
| ≥ 15% | 93.7 | 37.9 | 40.2 | 93.1 | 1.5 |
| ≥ 20% | 89.1 | 52.0 | 45.2 | 91.4 | 1.9 |
| ≥ 25% | 80.2 | 62.8 | 49.0 | 87.7 | 2.2 |
| ≥ 30% | 71.4 | 70.1 | 51.5 | 84.7 | 2.4 |
| ≥ 35% | 63.4 | 77.6 | 55.7 | 82.7 | 2.8 |
| ≥ 40% | 50.0 | 83.6 | 57.5 | 79.0 | 3.0 |
| ≥ 45% | 42.4 | 87.9 | 60.8 | 77.4 | 3.5 |
| ≥ 50% | 38.2 | 90.7 | 64.5 | 76.7 | 4.1 |
| ≥ 55% | 32.8 | 93.5 | 69.0 | 75.8 | 5.0 |
| ≥ 60% | 27.3 | 95.1 | 71.4 | 74.6 | 5.6 |
| ≥ 65% | 23.1 | 96.1 | 72.4 | 73.7 | 5.9 |
| ≥ 70% | 18.9 | 97.8 | 78.9 | 73.0 | 8.6 |
| ≥ 75% | 15.1 | 98.5 | 81.8 | 72.2 | 10.1 |
| ≥ 80% | 11.3 | 98.9 | 81.8 | 71.5 | 10.3 |
| ≥ 85% | 4.2 | 99.8 | 90.9 | 70.1 | 21 |
| ≥ 90% | - | - | - | - | - |
| ≥ 95% | - | - | - | - | - |
| ≥ 100% | - | - | - | - | - |
| Abbreviations: PPV, positive predictive value; NPV, negative predictive value; LR+, positive likelihood ratio (calculated using the equation: sensitivity/1-specificity).  ^a^ Predicted probability of having ≥5-year OS. | | | | | |

**Supplementary figures**


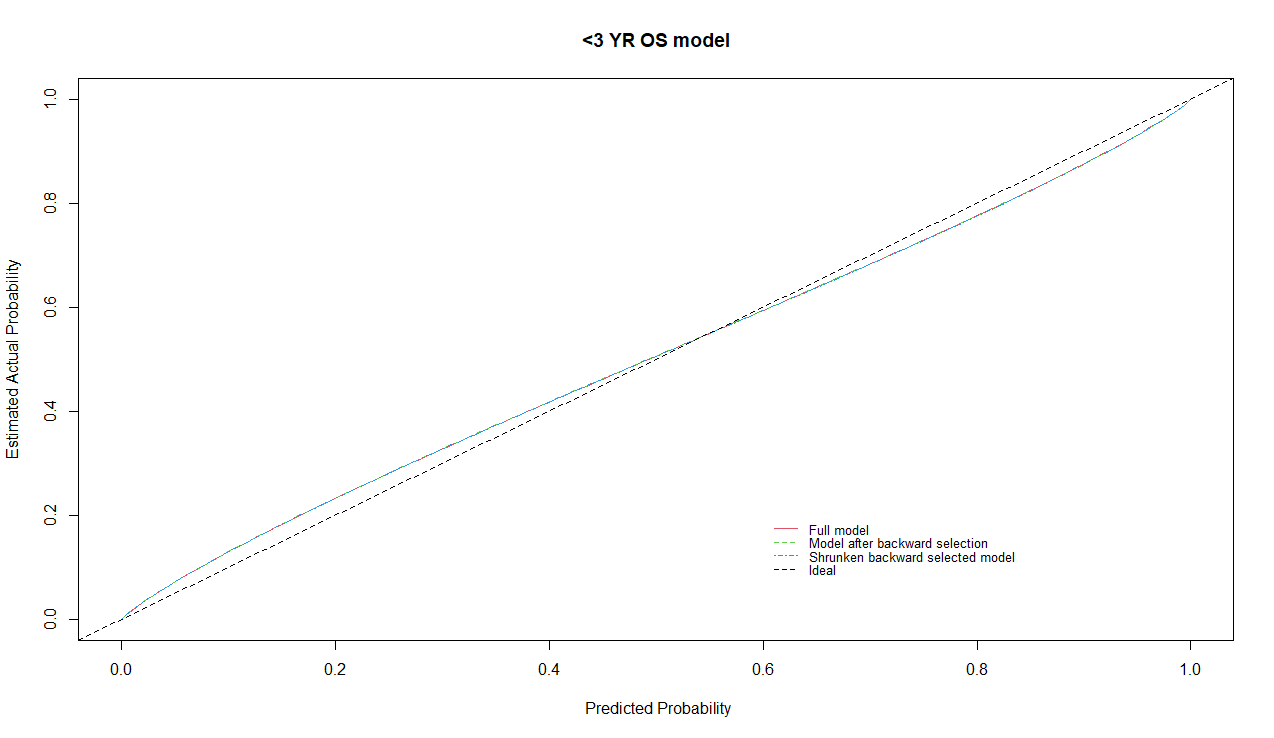


**Supplementary figure S1**. Calibration plot of the ≤3-year OS model before and after internal validation. The ideal line represents the perfect fit line. The model after backward selection represents the model before internal validation (green dotted line). The shrunken backward selection model represents the model after internal validation (blue line). The full model represents the model with all the candidate predictors (red line). The calibration plot demonstrates that the final ≤3-year OS model is well-calibrated.


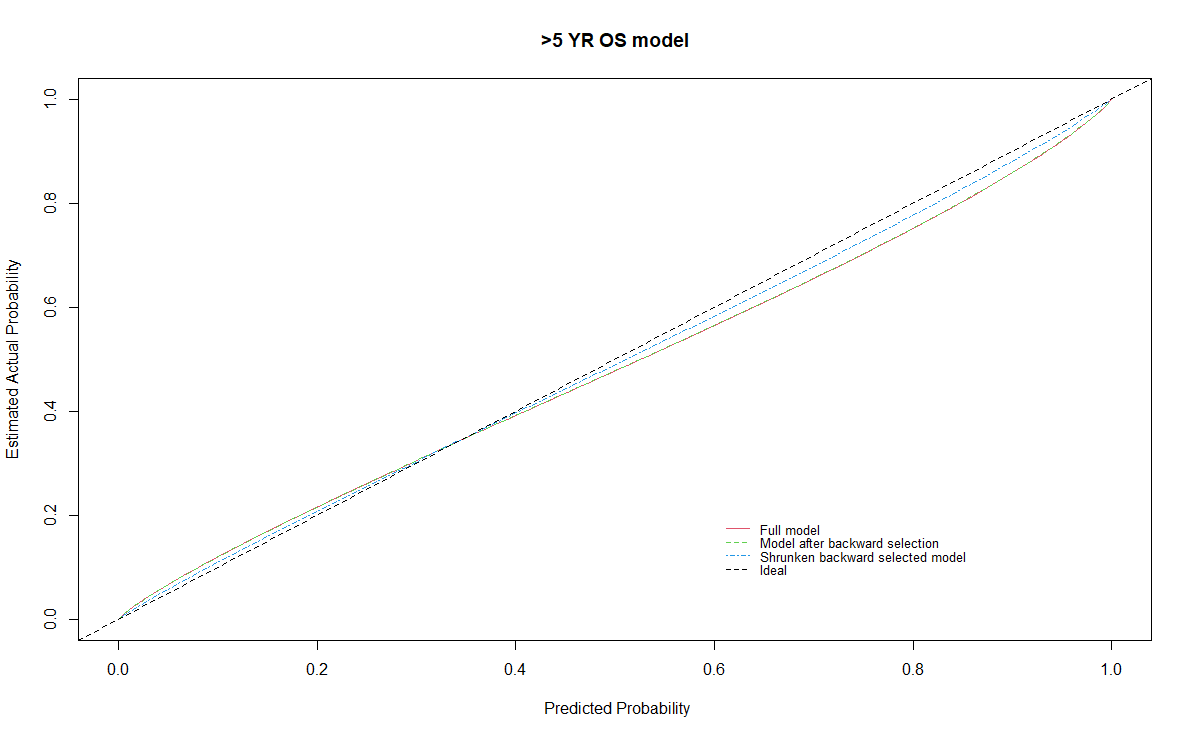


**Supplementary figure S2**. Calibration plot of the ≥5-year OS model before and after internal validation. The ideal line represents the perfect fit line. The model after backward selection represents the model before internal validation (green dotted line). The shrunken backward selection model represents the model after internal validation (blue line). The full model represents the model with all the candidate predictors (red line). The calibration plot demonstrates that the final ≥5-year OS model is well-calibrated.


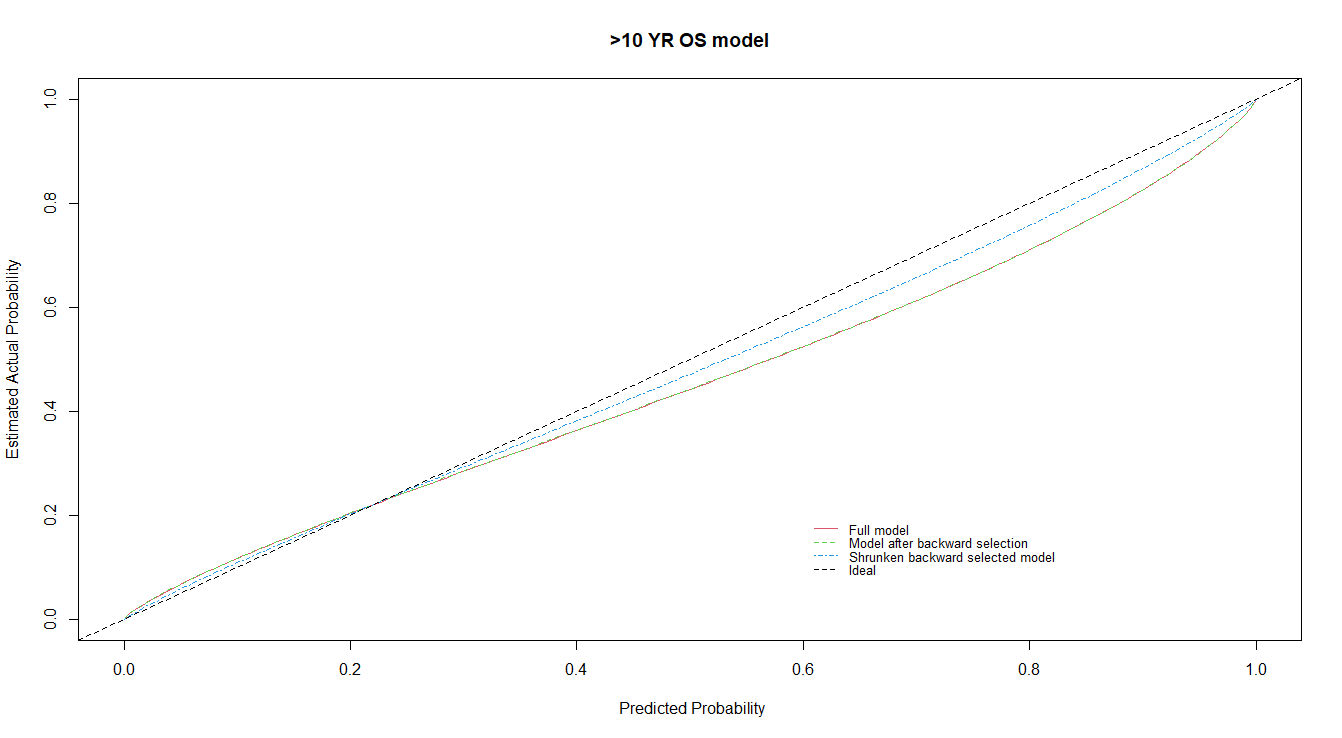


**Supplementary figure S3**. Calibration plot of the ≥10-year OS model before and after internal validation. The ideal line represents the perfect fit line. The model after backward selection represents the model before internal validation (green dotted line). The shrunken backward selection model represents the model after internal validation (blue line). The full model represents the model with all the candidate predictors (red line). The calibration plot demonstrates that the final ≥10-year OS model is well-calibrated.


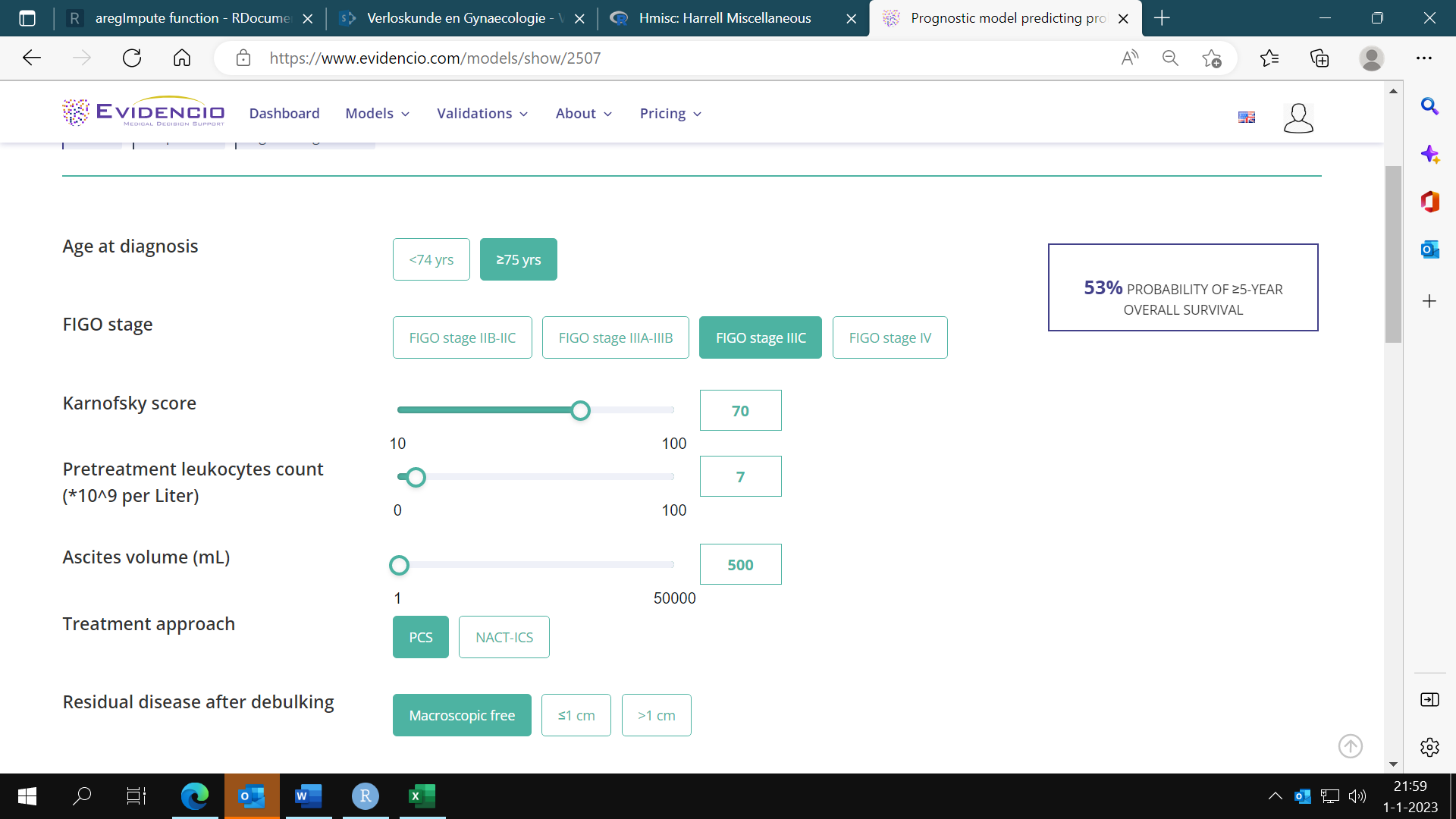


**Supplementary figure S4.** Screenshot of the online score calculator for the ≥5-year OS model.

The online score calculator allows clinicians to estimate the probability of ≥5-year OS. For example: a 81-year-old patient with FIGO stage IIIC EOC, who presented with Karnofsky score of 70, pretreatment leukocytes count of 7 x10^9^ per liter, 500 ml of ascites volume, who underwent primary cytoreductive surgery and complete cytoreduction. The nomogram predicts a probability of 53% of ≥5-year OS for this patient.
